# Rainfall- and Temperature-Driven Emergence of Neural Angiostrongyliasis in Eastern Australia, 2020–2024

**DOI:** 10.1093/infdis/jiaf173

**Published:** 2025-04-03

**Authors:** Phoebe Rivory, Rogan Lee, Michael P Ward, Jan Šlapeta

**Affiliations:** Sydney School of Veterinary Science, Faculty of Science, The University of Sydney, Sydney, New South Wales, Australia; New South Wales Health Pathology, Centre for Infectious Diseases and Microbiology Laboratory Services, Level 3 Institute of Clinical Pathology and Medical Research (ICPMR), Westmead Hospital, Westmead, New South Wales, Australia; School of Biomedical Sciences, Faculty of Medicine and Health, The University of Sydney, Sydney, New South Wales, Australia; Sydney School of Veterinary Science, Faculty of Science, The University of Sydney, Sydney, New South Wales, Australia; Sydney School of Veterinary Science, Faculty of Science, The University of Sydney, Sydney, New South Wales, Australia; Sydney Infectious Diseases Institute, The University of Sydney, Sydney, New South Wales, Australia

**Keywords:** neuroangiostrongyliasis, climate, canine, qPCR, ELISA

## Abstract

Neural angiostrongyliasis (NA), caused by rat lungworm (*Angiostrongylus cantonensis*), is an emerging zoonotic disease on Australia's east coast. The number of cases has risen since 2010. This study investigated the diagnosis, genetic diversity of *A cantonensis*, and spatial and temporal dynamics of canine NA (CNA). We analyzed cerebrospinal fluid samples from 180 clinically suspected cases (2020–2024) using AcanR3990 quantitative polymerase chain reaction, confirming infection in 93. Cases were detected around Brisbane and Sydney, with peak occurrence in 2022 (32 cases). Generalized linear modeling demonstrated that CNA occurrence depends on immediate and long-term rainfall (1- and 10- to 12-month lags) and medium-term temperature changes (5- to 7-month lags). Partial *cox*1 sequencing revealed Ac13 as the dominant haplotype (9/15). Comparison with an established enzyme-linked immunosorbent assay using 50 randomly selected samples showed substantial agreement (κ = 0.66). With many cases likely remaining undiagnosed, NA poses an ongoing One Health issue in Australia.

## Teaser

Rising canine neural angiostrongyliasis (CNA) cases along Australia's east coast signal growing health risks for humans and animals. Rainfall and temperature drive CNA seasonality, aiding evidence-based clinical suspicion and disease prediction.

## Graphical Abstract

**Graphical Abstract:**
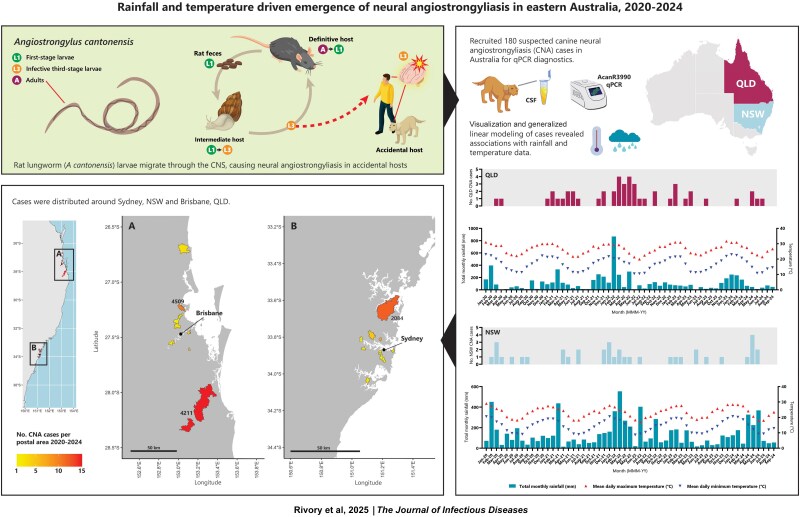

*Angiostrongylus cantonensis*, commonly known as the “rat lungworm,” is a nematode parasite recognized as the primary cause of neural angiostrongyliasis (NA), which is syndromically diagnosed as eosinophilic meningitis in humans [1–3]. The geographical distribution of *A cantonensis* spans the Asia-Pacific region and has recently expanded to include the United States and Europe [4–7]. The parasite follows a complex transmission cycle involving gastropods (snails and slugs) as intermediate hosts and rats as definitive hosts. Infective third-stage larvae (L3s) develop within gastropods and are then ingested by rats, where they mature into adults in the pulmonary arteries [8]. Rats excrete first-stage larvae in their feces, which are then ingested by gastropods to complete the cycle [8]. Transmission of L3s to “accidental” (aberrant) hosts, such as humans and dogs, occurs through ingestion of infected gastropods, or L3s released into the environment, which can potentially contaminate water sources [9]. Environmental factors that influence intermediate host activity, such as temperature and precipitation, ultimately influence the dispersal of L3s in the environment to both accidental and definitive hosts [10, 11]. Infective larvae cause NA when they migrate through the central nervous system (CNS) of accidental hosts including humans and dogs [1, 2, 8, 12]. The accidental hosts' immune response to *A cantonensis* in the CNS results in neurological manifestations caused by inflammation of the meninges and cerebral tissue, which can be fatal [12]. In Australia, where human NA was first recognized >50 years ago, dogs serve as a critical sentinel population for human NA, experiencing significantly higher infection rates that provide early warning of emerging human disease risks [13–16].

Canine neural angiostrongyliasis (CNA), the canine equivalent of human NA, is characterized by a range of debilitating signs including hindlimb and tail paralysis, urinary incontinence, and hyperesthesia [14, 15]. CNA has been reported sporadically along the coastal areas of Queensland (QLD) and eastern New South Wales (NSW) since 1972 [14, 15, 17]. and 2 *A cantonensis cox*1 haplotypes (Ac13 and SYD.1) have been identified as causative agents [18, 19]. A closely related Australian native species, *Angiostrongylus mackerrasae* (which is also neurotropic), has yet to be linked with infection in accidental hosts—aside from 1 case in a flying fox [20]—warranting species identification in cases. CNA demonstrates a seasonal pattern, with peak incidence in autumn [14, 15]. This seasonality aligns with periods of optimal temperature and moisture conditions for gastropod activity following rainfall events. The mortality rate ranges from 14% to 58%, and the most recent update on CNA in Australia revealed an increasing number of cases from 2010 to 2020 [13], suggesting escalating human health threats [21].

Diagnostics for NA remain challenging in both dogs and humans due to the rapid onset of the clinical signs and symptoms [1, 22, 23]. Laboratory diagnostics rely on the application of antibody detection or DNA amplification tests, with the introduction of AcanR3990 quantitative polymerase chain reaction (qPCR) assay demonstrated to be superior to previously used polymerase chain reaction (PCR)–based assays [13, 24].

Given the availability of canine cerebrospinal fluid (CSF) from Australian veterinarians for CNA diagnostics, we aimed to utilize the AcanR3990 qPCR assay to determine the spatial and temporal dynamics trends of the infection in dogs during the past 5 years, the causative *Angiostrongylus* species and haplotype, and potential associations between the cases and weather. Additionally, we compared the performance of the AcanR3990 qPCR assay with traditional enzyme-linked immunosorbent assay (ELISA) for diagnosing NA. The study enabled us to demonstrate the increased occurrence of CNA in Australia.

## MATERIALS AND METHODS

### Ethics Statement

The use of residual clinical samples was in accordance with the University of Sydney’s Animal Ethics Committee Protocol (2023/2278).

### Suspected CNA Case Recruitment

Material was provided for the purpose of molecular diagnostics requested by the veterinary practitioner from February 2020 to September 2024. CSF samples were submitted specifically due to clinical suspicion of CNA, and were collected by registered veterinary practitioners from around Australia. Samples were submitted to the Sydney School of Veterinary Science, the University of Sydney. CSF (and/or DNA from CSF) from 180 dogs were provided by Vetnostics (Laverty Pathology; North Ryde Laboratory, Sydney, Australia) and Veterinary Pathology Diagnostics Services (University of Sydney). DNA from CSF samples was isolated and eluted to 75 µL as previously described [18].

### Detection of *Angiostrongylus* in Canine CSF via AcanR3990 qPCR

A total of 180 DNA samples were isolated from canine CSF. DNA and remaining CSF were stored at −20°C. A probe-based qPCR assay targeting a repeat sequence on contig 3990 (AcanR3390) of *A cantonensis* developed was locally optimized [18, 24]. AcanR3390 qPCR amplifies both *A cantonensis* and *A mackerrasae* DNA [24]. Each sample was run in duplicate. To confirm successful DNA isolation, the AcanR3990 qPCR was run alongside qPCR for mammalian glyceraldehyde 3-phosphate dehydrogenase (G3PDH) gene [25]. Reagent concentrations and cycling conditions were kept consistent with our previous publication [18]. The qPCR assays were performed in a CFX96 Touch Real-Time PCR Detection System (Bio-Rad Laboratories, Inc) and cycle threshold (Ct) values were recorded using CFX Maestro Software 2.3 (Bio-Rad) with thresholds auto-calculated [18].

Samples were considered for further analysis if the G3PDH qPCR was positive (Ct <40) for both duplicates while the extraction control was negative for both G3PDH and *Angiostrongylus* (Ct ≥40). All samples passed this step. As with previous methods for qPCR results interpretation [18], samples were sorted into 4 positive categories and 1 negative category: “strong positive,” “positive,” “weak positive,” “equivocal,” and “negative” according to duplicate Ct values (Table 1).

**Table 1.** Results Interpretation for Canine Cerebrospinal Fluid DNA Samples Screened for *Angiostrongylus* via AcanR3990 Quantitative Polymerase Chain Reaction in Duplicate

AcanR3990 qPCR Duplicate Ct Values	Interpretation	No. of Samples	Total
Both Cts ≤35	Strong positive	75	93
1 Ct ≤35 and 1 Ct >35 to ≥40 (no amplification)	Positive	7	
Both Cts >35 to ≤38	Weak positive	11	
1 Ct >35 to <40 and 1 Ct >38 to ≥40 (no amplification)	Equivocal	16	87
Both Cts ≥40 (no amplification)	Negative	71	

Abbreviations: Ct, cycle threshold; qPCR, quantitative polymerase chain reaction.

### Visualization of Trends in CNA Cases Over Time and Space

AcanR3990 qPCR-positive samples (= cases) were mapped according to the submitting clinics' postcode in R version 4.1.2 (r-project.org) using the ggplot package (ggplot2.tidyverse.org).

Recent temporal trends in CNA were reevaluated by plotting cases according to year and month. Historical data were included in CNA counts by year to assess temporal changes [13]. For analyses by month, only cases in this study with collection date data available (n = 93/95) were used. If collection date data were not available, collection year was populated with the year the sample was received. Cases by month and year were plotted GraphPad Prism.

To visualize CNA seasonal trends, and to explore the environmental influence on *A cantonensis* transmission, the number of cases was plotted with rainfall and temperature data. As cases only occurred around the cities of Sydney (NSW) and Brisbane (QLD), weather data (monthly, 2020–2024; total rainfall [mm], mean daily maximum temperature [°C], and mean daily minimum temperature [°C]) from these locations were downloaded from the Australian Bureau of Meteorology climate data site (www.bom.gov.au/climate/data/). Due to missing data in total monthly rainfall for both Brisbane and Sydney, averages were calculated across 2 weather stations: 040913 and 040976 for Brisbane, and 066006 and 66214 for Sydney. Mean minimum and maximum daily temperature data were downloaded from stations 66214 and 040913 for Sydney and Brisbane, respectively. Climate data and CNA case numbers were plotted in GraphPad Prism separately according to their state.

### Time-Series Analysis to Determine Potential Associations Between CNA and Weather

To statistically examine the relationships between increased CNA occurrence and environmental conditions, a time-series analysis was conducted on monthly cases of CNA in QLD and NSW, Australia and associated climate variables (rainfall and mean temperature) from February 2020 to September 2024. The Brisbane (QLD) and Sydney (NSW) data were averaged and analyzed together to increase statistical power. To observe seasonal trends in the case data, a smooth trendline plot using locally estimated scatterplot smoothing and a decomposition plot were produced. Data stationarity was assessed using Augmented Dickey-Fuller tests, with first-order differencing applied to nonstationary series (total cases and rainfall; *P* = .06 and *P* = .10, respectively). Cross-correlation functions were computed to examine relationships between climate variables and CNA cases at different time lags (up to 12 months). Generalized linear regression models were developed to assess the relationship between differenced CNA cases and lagged climate variables. Four modeling approaches were employed: univariate models for rainfall and temperature separately, a combined model using only significant variables from univariate analyses, and 3 comprehensive models incorporating all variables with up to 6-, 10-, and 12-month lags. Model selection was performed using stepwise regression with bidirectional elimination. Model diagnostics included assessment of residual autocorrelation using Durbin-Watson tests, checks for stationarity, and visual inspection of residual plots. Model fit was evaluated using adjusted *R*^2^ values, and model selection involved consideration of both model fit and biological plausibility.

### Molecular Determination of *Angiostrongylus* Species and *cox*1 Haplotype

To determine the species and *cox*1 haplotype of *Angiostrongylus,* a 206-bp *cox*1 region (excluding primer sequence) was amplified and sequenced according to “assay 2” described previously [19]. All AcanR3990 qPCR-positive samples were subjected to *cox*1 assay; however, due to low concentration of target *Angiostrongylus* DNA in CSF, only samples with a “strong positive” result were considered for subsequent PCRs. The *cox*1 qPCR reactions were run on the CFX96 Touch Real-Time PCR Detection System with thresholds auto-calculated. Results were considered positive if Ct <38 and the melt curve profile corresponded to that of the *A cantonensis* control. PCR products were bidirectionally sequenced at Macrogen Inc (Seoul, Korea). The DNA chromatographs were visually inspected in CLC Main Workbench v22 (Qiagen, CLC bio). Sequences were matched against a compilation of *cox*1 sequences from GenBank, and a phylogenetic tree was constructed in CLC Main Workbench v22 (Supplementary Figure 1).

### Comparison of AcanR3990 qPCR Results to Traditional Antibody Detection in CSF

A random selection of raw CSF samples (n = 50; 35 positive, 11 negative, and 4 equivocal for AcanR3990 qPCR) was processed for the *A cantonensis* antibody detection using an ELISA adopted for use in diagnostics and research at Westmead Hospital [14, 26, 27]. In brief, soluble antigens from adult whole male and female *A cantonensis* (1:1 ratio) were coated onto 96-well MaxiSorb plates (Thermo Fisher Scientific, Australia) at a concentration of 5 µg/mL. Dog CSF samples were diluted in a 2-fold serial dilution from 1:100 to 1:12 800 in phosphate-buffered saline (PBS) containing 0.2% milk powder (Difco Skim Milk; BD, Macquarie Park, Australia) and 0.05% Tween 20. A positive CSF control was included on each plate, with serial dilutions performed in the same manner as unknown samples. At least 1 negative CSF control was included, with 4 replicates at a 1:100 dilution to determine titer cut-off values. Samples were incubated in the coated plates at 37°C for 1 hour in a humidity chamber. The secondary antibody, rabbit anti-dog immunoglobulin H conjugated to horseradish peroxidase (Sigma, St Louis, Missouri; diluted to 1:16 000), was allowed to incubate at 37°C for 1 hour in a humidity chamber. Both incubations were followed by 3 washes with PBS containing 0.05% Tween 20. Tetramethylbenzidine substrate (GraphicScientific Pty Ltd, Australia) was allowed to react for 15 m in at room temperature in the dark, and stopped with 1 M phosphoric acid. Wells were read at 450 nm using an iMark Microplate Reader (Bio-Rad, Australia). Titration curves were plotted for visual examination, and the cut-off threshold was determined by taking the average optical density reading of the negative CSF controls plus 2 standard deviations. Samples were considered positive if at least the 1:100 dilution was greater than the cut-off, although samples with poor titer curves and close to the cut-off value were considered equivocal. Samples with no titer were considered negative.

To assess the performance of this ELISA in comparison to the AcanR3990 qPCR, a contingency table was created to compare positive/negative results. Sensitivity and specificity (%) were calculated with 95% confidence intervals (CIs) in GraphPad Prism (version 9.5.1, GraphPad Software, USA). The degree of agreement was quantified with the κ coefficient using GraphPad QuickCalcs (www.graphpad.com/quickcalcs/kappa1/) with 95% CI.

## RESULTS

### CNA Cases Peaked in 2022 and Infection Association With Rainfall Is Apparent

A total of 180 canine CSF samples were submitted due to clinical suspicion of CNA and were screened using *Angiostrongylus* AcanR3990 qPCR during February 2020–September 2024. According to the results interpretation criteria for AcanR3990 qPCR, 75 were strong positive, 7 were positive, 11 were weak positive, 16 were equivocal, and 71 were negative (Table 1). We confirmed 93 CNA cases with a combined average of duplicate Ct values of 31.28 (range, 24.42–39.76).

**Figure 1.**
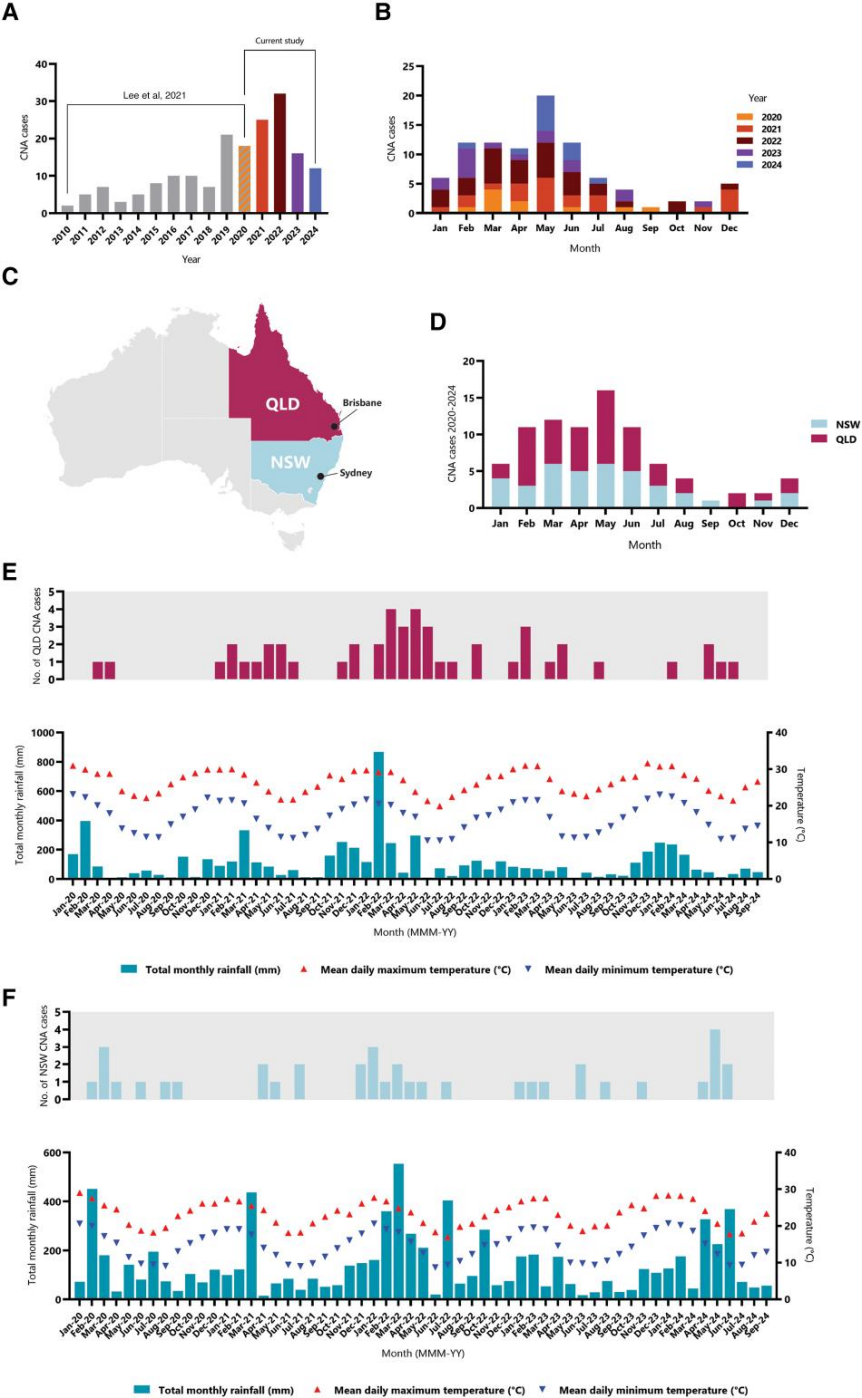
Seasonal and temporal trends of canine neural angiostrongyliasis (CNA) in Australia. *A*, Total count of CNA cases per year including data from 2020 to 2024 (this study) and previously from 2010 to 2020 [13]. *B*, Total count of CNA cases from 2020 to 2024 (with collection date data), diagnosed by AcanR3990 quantitative polymerase chain reaction on cerebrospinal fluid DNA. Cases are plotted by month. *C*, Simplified map of Australia showing the 2 states where positive CNA cases were detected: New South Wales (NSW) and Queensland (QLD). The major cities, Sydney and Brisbane, are labeled. *D*, Total count of CNA cases (with clinic location data) per month over the 2020–2024 period according to the state of origin (NSW and QLD). *E*, Count of CNA cases recorded in QLD per month aligned with weather data obtained from stations in Brisbane (station numbers 040913 and 040976) from the Australian Bureau of Meteorology climate data site (BOM, www.bom.gov.au/climate/data/). Total monthly rainfall (mm) is plotted as bars on the left y-axis, and mean daily maximum temperature (°C; red triangles) and mean daily minimum temperature (°C; blue triangles) are plotted on the right y-axis. *F*, Count of CNA cases recorded in NSW per month aligned with weather data obtained from stations in Sydney (station numbers 066006 and 66214) from BOM. Total monthly rainfall (mm) is plotted as bars on the left y-axis, and mean daily maximum temperature (°C; triangles) and mean daily minimum temperature (°C; inverted triangles) are plotted on the right y-axis.

The total number of CNA cases per year from 2010 to 2020 [13] and from 2020 to 2024 (current study) revealed a gradual upward trend, with the highest number of cases (n = 32) occurring in 2022 (Figure 1*A*). In the most recent 2 years (2023–2024), CNA cases decreased substantially, but not to numbers observed prior to 2019 [13]. Most cases from 2020 to 2024 generally occurred in May and the least in September (Figure 1*B*).

Of the 8 states and territories of Australia, cases only occurred in NSW and QLD (Figure 1*C*). The seasonal trend in cases from both NSW and QLD between 2020 and 2024 was similar (Figure 1*D*); however, in September, cases were from NSW only and in October, cases were from QLD only. There also appeared to be a more dramatic spike in QLD cases in the months of February and May.

Plotting of climatic variables (monthly; total rainfall [mm], mean daily maximum temperature [°C], and mean daily minimum temperature [°C]) from 2020 to 2024 was suggestive of CNA's association with decreasing temperatures and higher rainfall (Figure 1*E* and 1*F*). There was also evidence of a lag effect from recent historical rainfall occurring a few months prior to the seasonal spikes in CNA rates (Figure 1*E*).

### Confirmed Cases Surrounded the Cities of Sydney and Brisbane

**Figure 2.**
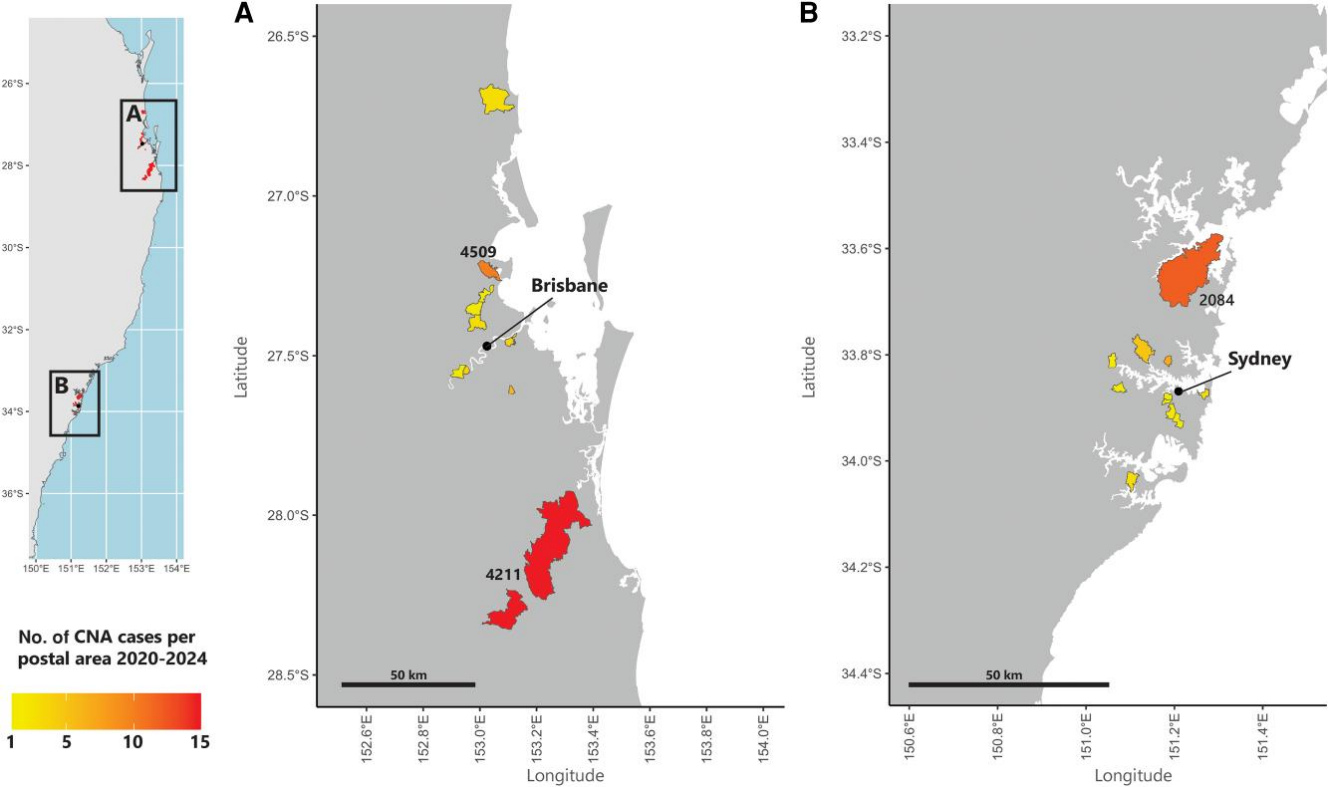
Map of canine neural angiostrongyliasis (CNA) cases in eastern Australia by postal area from 2020 to 2024 determined via AcanR3990 quantitative polymerase chain reaction applied to cerebrospinal fluid DNA. Cases were restricted to the eastern coast of Australia surrounding Brisbane, Queensland (inset *A*) and Sydney, New South Wales (inset *B*). The total number of cases (indicated by the yellow-red color gradient) ranged between 1 and 15 per postal area. Map of eastern Australia with showing postcodes showing the frequency of detected canine neural angiostrongyliasis (CNA), providing focused areas of postcodes around Brisbane (*A*) and Sydney (*B*).

Suspected CNA CSF samples with clinic addresses provided (n = 169) were received from a total of 29 unique Australian postal areas. Confirmed cases (with clinic addresses provided, n = 86) originated from a total of 21 Australian postal code areas (11 in NSW and 10 in QLD). CNA cases were restricted to the eastern coast of Australia, near Brisbane, QLD and Sydney, NSW (Figure 2). The total number of cases ranged between 1 and 15 per postal area over the 4-year period, with higher case numbers (≥10) appearing in 3 larger postal areas; 1 in NSW (2084, Terrey Hills area) and 2 in QLD (4211, Gold Coast area and 4509, North Lakes area).

### Time-Series Analysis Reveals Lag Effect of Climatic Data

The 2 best-performing models were the 10-month lag stepwise model and the 12-month lag stepwise model. The former revealed significant associations between CNA cases and temperature at lags 2, 3, 5, 6, and 7 months, with total monthly rainfall significant at lag 10 months (Table 2). The more comprehensive 12-month lag stepwise model demonstrated improved model fit, identifying significant relationships with rainfall at lags 0, 1, 2, 11, and 12 months, and temperature at lags 2, 3, 5, 6, 7, and 10 months (Table 2). Both models passed diagnostic tests for autocorrelation and stationarity (Table 2). The higher adjusted *R*² of the 12-month lag stepwise model suggests that including longer-term lagged effects (up to 12 months) provides better explanatory power, but this may be at the cost of model parsimony (Table 2). Cross correlation function, decomposition, and model diagnostic plots are shown in Supplementary Figure 2.

**Table 2.** *Angiostrongylus* Antibody Enzyme-Linked Immunosorbent Assay Titers, and Results Interpretation for a Subset (n = 50) of Canine Cerebrospinal Fluid Samples

Antibody ELISA Titer	Interpretation	No. of Samples	Total
1:100	Positive	9	35
1:200		10	
1:400		5	
1:800		3	
1:1600		1	
1:3200		0	
1:6400		0	
>1:12 800		7	
1:100, but poor curve	Equivocal	1	15
NA	Negative	14	

Abbreviations: ELISA, enzyme-linked immunosorbent assay; NA, <1:100 (starting dilution of CSF).

### Partial *cox*1 Sequencing Revealed Most Cases Are Implicated by the Ac13 Haplotype

Of the 93 cases confirmed by AcanR3990 qPCR and subjected to the partial *cox*1 qPCR, 34 *cox*1 amplicons were submitted for Sanger sequencing and 44% (15/34) were successfully sequenced. All 15 were matched *A cantonensis cox*1 sequences; Ac13 haplotype was identified in 9 of 15 (60% [95% CI, 35.75%–80.18%]) and the remaining 6 samples matched the SYD.1 haplotype.

### AcanR3990 qPCR Detects More Cases Than Antibody Detection in CSF During Acute Onset

**Figure 3.**
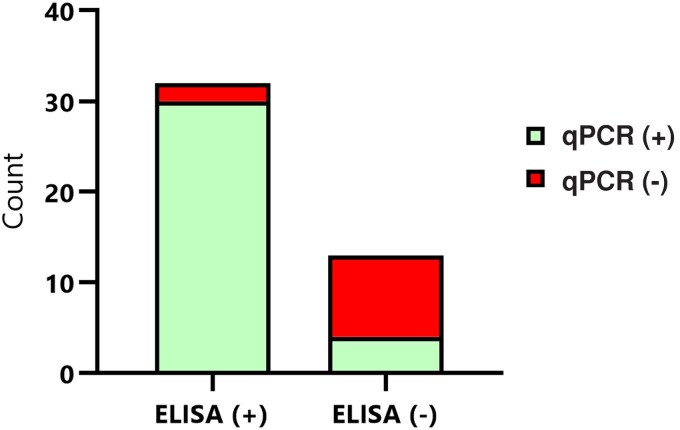
Contingency analysis of the performance of AcanR3990 quantitative polymerase chain reaction (qPCR) and antibody enzyme-linked immunosorbent assay (ELISA) for detecting *Angiostrongylus* infection in canine cerebrospinal fluid samples. Bar chart graph showing proportion of *Angiostrongylus cantonensis* ELISA positive and negative samples using diagnostic qPCR.

Of the 50 randomly selected samples where spare raw CSF was available, 35 were positive, 14 were negative, and 1 was equivocal according to the ELISA assay. Positive titers ranged from 1:100 to beyond 1:12 800 (Table 3). After excluding equivocal and negative results, contingency tables revealed that 30 of 50 samples were positive for both AcanR3990 qPCR and ELISA, 4 samples were positive via AcanR3990 qPCR only, 2 samples were positive via ELISA only, and 9 were considered negative in both assays (Figure 3). The Kappa agreement was substantial according to the Landis and Koch scale (κ = 0.66 [95% CI, .412–.908]). Using the AcanR3990 qPCR assay as the gold standard, the specificity of the ELISA was 88.24% (95% CI, 73.38%–95.33%) and the sensitivity was 81.82% (95% CI, 52.30%–96.77%).

**Table 3.** Model Fit and Coefficients From Multiple Linear Regression Modeling of Monthly Canine Neural Angiostrongyliasis Cases With Rainfall and Mean Temperature

Variable	12-Month Lag Stepwise Model		10-Month Lag Stepwise Model	
	Coefficient	*P* Value	Coefficient	*P* Value
Intercept	2.22	.83	**−**9.96	.02*
Rainfall	0.00	.09	…	
1-month lag rainfall	0.01	.01*	0.00	.18
2-month lag rainfall	0.00	.05	…	
10-month lag rainfall	…		0.00	.04*
11-month lag rainfall	0.00	.01*	…	
12-month lag rainfall	0.00	.01*	…	
2-month lag temperature	0.53	.03*	0.62	.00*
3-month lag temperature	**−**0.67	.01*	**−**0.57	.01*
5-month lag temperature	1.09	.00*	1.02	.00*
6-month lag temperature	**−**1.75	.00*	**−**1.52	.00*
7-month lag temperature	1.04	.00*	0.94	.00*
10-month lag temperature	**−**0.36	.07	…	
Model statistics				
Adjusted *R*^2^	0.50		0.43	
F-statistic	4.93 (11, 32 *df*)		5.78 (7, 38 *df*)	
*P* value	<.001*		<.001*	
Post hoc tests on model residuals				
Augmented Dickey-Fuller test for stationarity (*P* value)	<.01*		.018*	
Durbin-Watson test for autocorrelation (*P* value)	.76		.69	

The 2 best-performing models (12-month lag stepwise model and 10-month lag stepwise model) are shown.

Abbreviation: *df*, degrees of freedom.

^*^Significant (*P* < .05).

## DISCUSSION

Yearly CNA cases peaked in 2022, followed by a substantial decrease in the subsequent 2 years, though not returning to pre-2019 levels observed in the decade before [13]. In this study, we wanted to explain the apparent increase and peak of CNA. The extremely wet weather in 2022 [28] likely contributed to this spike, as rainfall encourages snail and slug (gastropods are *A cantonensis* intermediate hosts) proliferation and thus availability of infective larvae for rats and accidental hosts [10, 11, 29, 30]. This hypothesis is supported by the positive regression coefficients in our model for recent rainfall (up to 2 months prior), but importantly, longer-term rainfall (10–12 months prior) suggests a cumulative environmental conditioning effect [31]. In both selected models, alternating positive and negative temperature coefficients were seen in midterm lags (2–10 months prior). These oscillating temperature coefficients are indicative of complex interactions between the parasite and its multiple hosts. The timing of these oscillations may relate to the patent period in rats (∼44 days), the development time in gastropods (∼17–30 days), and the incubation period in dogs (∼9–14 days) [12, 32–35]. Nevertheless, temperature does largely influence larval development times and parasite release from gastropods [36, 37], desiccation rates (of gastropods and rat feces), and overall activity of these hosts [11]. Enriching these models with abundance of the intermediate hosts could be valuable for predicting high-risk periods for CNA transmission, especially outside of CNA's regular seasonal fluctuations.

Cases were confirmed predominantly in and around the cities of Sydney and Brisbane, likely due to access to emergency veterinary care and higher population density in these metropolitan areas. Several suburban postcodes experienced higher CNA occurrence, possibly due to denser vegetation supporting rat and gastropod populations [38–40]. A key constraint in our spatial analyses was the use of the veterinary clinic location rather than the dog's actual residence for georeferencing, which may have introduced sampling bias [41]. However, considering the high population densities of these areas and emergency nature of case presentation, such bias is not expected to be substantive. Additionally, the absence of reliable population-at-risk estimates and uncertainty around precise infection timing prevented us from employing more complex statistical methods such as Poisson regression. While a finer spatial resolution would enable more detailed modeling of CNA cases, our current dataset lacks this granularity. Despite these limitations, our approach successfully identified climate relationships with minimal assumptions about population structure and infection timing.

Molecular analysis of the dogs' CSF through *cox*1 sequencing identified only the invasive *A cantonensis.* While this might suggest that the Australian native *A mackerrasae* plays a minimal role in CNA, it would be premature to dismiss its potential involvement [23]. The known distribution of *A mackerrasae* largely overlaps with *A cantonensis* [8, 42–44]. It is possible that *A mackerrasae* is not present in Sydney, NSW, due to its preferred (native) rat hosts typically not persisting in urban environments—especially after their extirpation in the early 1900s [45]. For both Sydney and Brisbane, perhaps competition from more pervasive species (eg, black rat [*Rattus rattus*]), which typically harbor *A cantonensis*, has driven out the preferred hosts of *A mackerrasae* [45–47]. Subspecies haplotype determination of *A cantonensis* showed a predominance of the Ac13 haplotype over the SYD.1 haplotype. Combining this with *cox*1 sequence data from the cases reported previously [19], the total number of CNA cases with Ac13 detected is 18 of 25 (72% [95% CI, 52.42%–85.72%]). The overall majority of cases are caused by the Ac13 haplotype, which may be an indication of phenotypic differences between genetically distinct types [48].

The comparison between diagnostic methods confirms that while both the AcanR3990 qPCR and ELISA showed substantial agreement, the qPCR detected more cases [13]. The discrepancies observed may be attributed to delayed production of detectable antibodies [3], potential cross-reactivity with other pathogens [49], and the limited duration and quantity of the parasite in CSF [23, 50]. Notably, there were 2 AcanR3990 qPCR-negative samples that tested positive using the ELISA, both with titers >1:12 800. This may indicate that these canine patients were further into the convalescent period of the infection, where they have had time to clear parasite DNA from their CSF [3].

In conclusion, the study highlights the significant impact of environmental factors, particularly rainfall, on the occurrence of CNA in Australia. To enhance predictive models, future research should focus on incorporating data on gastropod abundance and the availability of infective larvae. Importantly, the findings from canine data and models are transferable to human disease risk, exemplifying the One Health approach to understanding and mitigating NA transmission dynamics.

## Supplementary Material

jiaf173_Supplementary_Data

## Supplementary Data

Supplementary materials are available at *The Journal of Infectious Diseases* online (http://jid.oxfordjournals.org/). Supplementary materials consist of data provided by the author that are published to benefit the reader. The posted materials are not copyedited. The contents of all supplementary data are the sole responsibility of the authors. Questions or messages regarding errors should be addressed to the author.

## Notes

***Acknowledgments*.** The authors thank Doug Hayward and Susan Jaensch from Vetnostics (Laverty Pathology; North Ryde Laboratory, Sydney, Australia) and Ignacia Meza and Christine Black from Veterinary Pathology Diagnostic Services (The University of Sydney) for providing the clinical samples for the study; Emily Francis for assisting with PCR assays; and the Parasitology group at the Sydney School of Veterinary Science for discussions during this study.

***Author contributions*.** P. R.: Conceptualization, methodology, formal analysis, investigation, data curation, writing–original draft, visualization, project administration. M. P. W.: Formal analysis, writing–review and editing. R. L.: Supervision, methodology, resources, project administration. J. Š.: Supervision, resources, methodology, writing–review and editing, project administration.

***Data availability*.** Newly obtained *cox*1 sequences were deposited in GenBank (accession numbers PQ660902–PQ660906). All data used in this study are available at LabArchives at https://dx.doi.org/10.25833/0aqd-jj85.

***Financial support***. This study was in part funded by the Betty and Keith Cook Canine Bequest Research Fund, Sydney School of Veterinary Sciences, The University of Sydney. P. R. was supported by The University of Sydney Research Training Program Tuition Fee Offset.
