# Supplementary material for: Rainfall- and Temperature-Driven Emergence of Neural Angiostrongyliasis in Eastern Australia, 2020–2024
Source: J Infect Dis. 2025 Apr 3;232(1):e150–8. doi: 10.1093/infdis/jiaf173 (PMC12308654; doi:10.1093/infdis/jiaf173)
Supplement: jiaf173_Supplementary_Data [file jiaf173_supplementary_data.zip › Supplementary_Figure_1.docx]

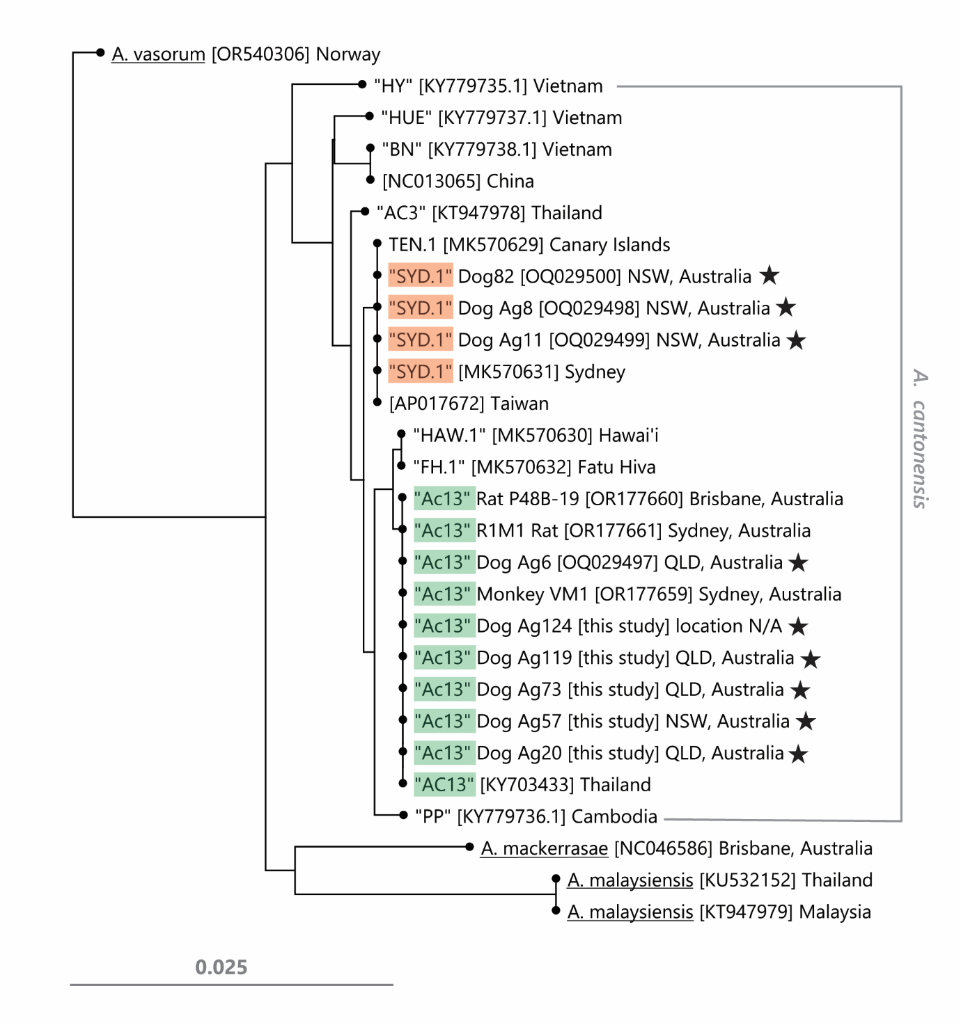


**Supplementary Figure 1** – Phylogeny of *A. cantonensis* partial *cox*1 sequences, including the nine sequenced in the current study (indicated by stars). GenBank numbers for each sequence have been provided in square brackets. The tree was constructed using the neighbour-joining method with Jukes-Cantor nucleotide substitution model and rooted with *Angiostrongylus vasorum* [OR540306]. The *cox*1 haplotypes in Australia are in green “Ac13” and orange “SYD.1”. The scale bar indicates the evolutionary distance.
