# Supplementary material for: Rainfall- and Temperature-Driven Emergence of Neural Angiostrongyliasis in Eastern Australia, 2020–2024
Source: J Infect Dis. 2025 Apr 3;232(1):e150–8. doi: 10.1093/infdis/jiaf173 (PMC12308654; doi:10.1093/infdis/jiaf173)
Supplement: jiaf173_Supplementary_Data [file jiaf173_supplementary_data.zip › Supplementary_Figure_2.docx]

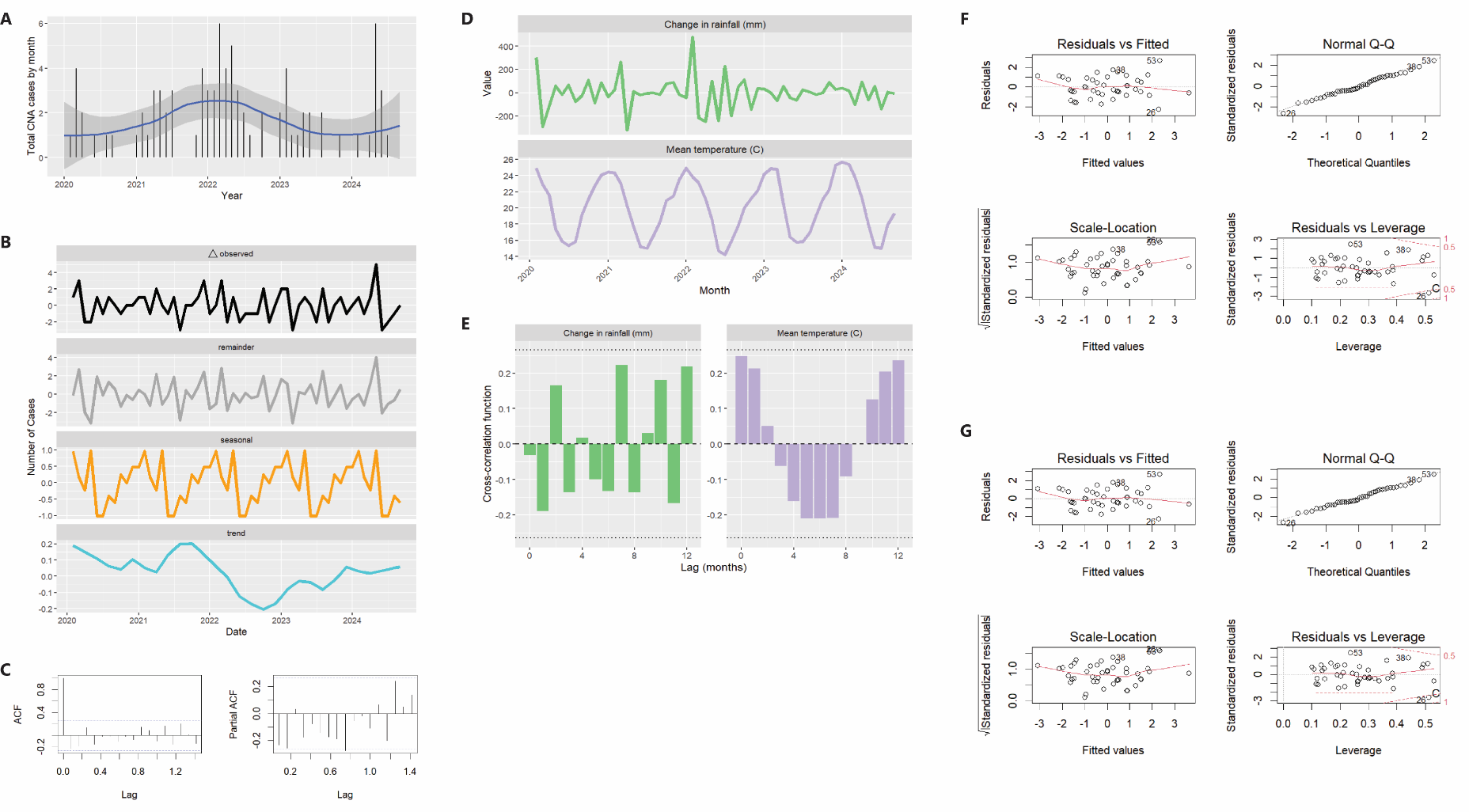


**Supplementary Figure 2.** Time series analysis of canine neural angiostrongyliasis (CNA) cases and predictive modelling. **(A)** Monthly CNA case counts with LOESS smoothed trend line illustrating temporal variation in disease incidence from 2020 to 2024. **(B)** Time series decomposition of differenced case series, showing observed data, trend, seasonal, and remainder components. **(C)** Autocorrelation Function (ACF) and Partial Autocorrelation Function (PACF) plots of differenced case series, demonstrating temporal dependencies and potential lag structures in the time series. **(D)** Time series of differenced predictor variables, displaying monthly changes in total rainfall (mm) and mean temperature (°C). **(E)** Cross-correlation function plots exploring temporal relationships between differenced rainfall, temperature, and CNA case series, identifying potential lagged interactions. **(F)** Diagnostic plots for the 12-month lag stepwise regression model, including residuals vs. fitted values, normal Q-Q plot, scale-location plot, and residuals vs. leverage to assess model assumptions and identify potential outliers or influential observations. **(G)** Identical diagnostic plots for the 10-month lag stepwise regression model to compare model performance and validate regression assumptions.
